# Proposed Classification of Auriculotemporal Nerve, Based on the Root System

**DOI:** 10.1371/journal.pone.0123120

**Published:** 2015-04-09

**Authors:** Iulian Komarnitki, Jacek Tomczyk, Bogdan Ciszek, Marta Zalewska

**Affiliations:** 1 Department of Descriptive and Clinical Anatomy, Medical University of Warsaw, Warsaw, Poland; 2 Department of Biological Anthropology, Cardinal Stefan Wyszynski University, Warsaw, Poland; 3 Department of Environmental Hazard Prevention and Allergology, Medical University of Warsaw, Warsaw, Poland; Duke University, UNITED STATES

## Abstract

The topography of the auriculotemporal nerve (ATN) root system is the main criterion of this nerve classification. Previous publications indicate that ATN may have between one and five roots. Most common is a one- or two-root variant of the nerve structure. The problem of many publications is the inconsistency of nomenclature which concerns the terms “roots”, “connecting branches”, or “branches” that are used to identify the same structures. This study was performed on 80 specimens (40 adults and 40 fetuses) to propose a classification based on: (i) the number of roots, (ii) way of root division, and (iii) configuration of interradicular fibers that form the ATN trunk. This new classification is a remedy for inconsistency of nomenclature of ATN in the infratemporal fossa. This classification system has proven beneficial when organizing all ATN variants described in previous studies and could become a helpful tool for surgeons and dentists. Examination of ATN from the infratemporal fossa of fetuses (the youngest was at 18 weeks gestational age) showed that, at that stage, the nerve is fully developed.

## Introduction

The auriculotemporal nerve (ATN) is a sensory branch of the mandibular nerve (MN), which is the third branch of the trigeminal nerve (TN) [1, 2]. Its branches innervate the temporal region, temporomandibular joint and the site of the auricle of the ear. In addition to the sensory fibers, ATN also carries the autonomic fibers. The autonomic fibers originate from the lesser petrosal nerve and middle meningeal artery plexus. ATN divides into numerous branches that supply the external ear and, after that, to the parotid gland and minor salivary glands of the cheek and lips [3]. ATN passes through the infratemporal fossa, retromandibular fossa, and the temporal region. ATN anatomy is commonly studied in the latter two locations [4–8] in contrast to a detailed ananlysis of the way of nerve root division and interradicular nerve connections in the infratemporal fossa region. The reason for this trend is the irregular shape of the cavity and limited access to ATN there. Access to the nerve at this site is difficult due to the many skeletal obstacles. The nerve is laterally enclosed by the ramus of the mandible, superiorly by the greater wing of the sphenoid and anteriorly by the infratemporal surface of the maxilla. The issue of ATN in the temporal region is well described in the literature [4, 8] whereas the detailed description of the root system, which is a part of the infratemporal portion of the nerve, is rarely a subject of research.

To date, ATN structure has only been examined in the context of the topography of the nerve trunk in respect to the temporomandibular joint [5, 9] or to the topographical relationship of the nerve roots and middle meningeal artery (MMA) [10,11]. Few papers considered the analysis of the root variations of ATN [10–14]. It is very important to note that all cited studies of ATN variation concern data from adults only, omitting data from the fetus population, and that the publication of Soni [13] is a mere case study. The knowledge of changes that occur within the infratemporal fossa over the course of ontogeny will enable to understand the mechanism concerning the development of craniofacial malformations and their consequences. Thus, our current knowledge of variations in the infratemporal fossa section of ATN in adult and fetus populations suggests the need for more in-depth research.

The aim of this study is to classify the variety of ATN structure in the infratemporal fossa. The proposed classification will be evaluated in the context of suitability in both adult and fetus populations.

## Materials and Methods

In the study presented here, adult and fetus corpses were used. All anatomical preparations used in the research came from donations to the Department of Descriptive and Clinical Anatomy, Medical University of Warsaw. Parents of children or fetuses whose remains were used in the study gave informed consent that they be used for scientific purposes.

According to the Polish law, a corpse donated for scientific or didactic activity in lieu of burial is initially transported to the university where it will be studied. All cadavers used in this study were presented as donations to the University of Warsaw. Each cadaver donation was properly documented and the documentation archived at the Department of Descriptive and Clinical Anatomy, University of Warsaw (Chalubiński 5 str; 02–004 Warsaw, Poland; https://anatomia.wum.edu.pl/index.php/en/).

No ethics committee/IRB approval was obtained because in Poland the approval of the ethical committee is necessary when investigation on living people is conducted or the identity of the human material is necessary for scientific or clinical purposes. In our investigation the material is anonymised. Furthermore, Polish law requires ethics committee or internal review board (IRB) approval when scientific investigation of living persons is conducted or when the identity of a deceased individual being used for scientific purposes is relevant to the study. This study involved only donated corpses whose identities were irrelevant to the study, therefore no IRB or ethics committee approvals were needed or obtained. The identities of the cadavers were never made available to the research team.

In Poland, human bodies used in a scientific study arrive at the study site by means of one of two legal avenues: the individual declares while alive and of sound mind that he or she wishes their corpse to be used for scientific advancement upon their death, or, in the absence of the deceased’s previously expressed intentions, a legal representative of the deceased has the right to direct the remains for scientific purposes as long as no legal heirs object to the decision to donate. The corpses used in this study arrived by both methods of donation. With absolute respect for the donations, all identifying data of the individuals under study and their families are and will remain concealed.

The research team used autopsy material obtained from the Department of Descriptive and Clinical Anatomy of the Medical University of Warsaw. A total of 80 cadaveric ATN specimens fixed in 4% formaldehyde solution were used in this study. There was no evidence of developmental pathology exhibited by any of the 80 specimens under study. Forty of the specimens were taken from 22 adult cadavers that are described as:

- 16 males, 6 females
- All between 57 and 85 years of age at time of death

Forty specimens taken from 22 fetus cadavers included:

- 7 males, 15 females
- Every fetus was between at 18 and 37 weeks of gestation at time of death

The analysis was conducted exclusively on the specimens that did not show pathology in development.

The following instruments were used: surgical instrument set, low-speed handpiece with a set of discs and cutters, microsurgical Chifa instrument set, stereomicroscope—Nikon SM 1500 with a possibility of examination with reflected and transmitted light (eyepiece: C-W 10X B/22, objective: WD 136 Nikon Japan HR Plan Apo 0,5X, magnification—from 0.75 to 11.25 [0.75, 1, 2, 3, 4, 5, 6, 7, 8, 9, 10, 11.25]), surgical loupes NY Dornwell 3,0X, metric-inch electronic calipers 150 mm; accuracy 0.02 mm, measurement 0.01 mm, microscopic measurement system NIS Elements D 4.0. Classical microanatomical preparation techniques were used to prepare ATN. The preparation technique was different in adults and fetuses.

**Fig 1:**
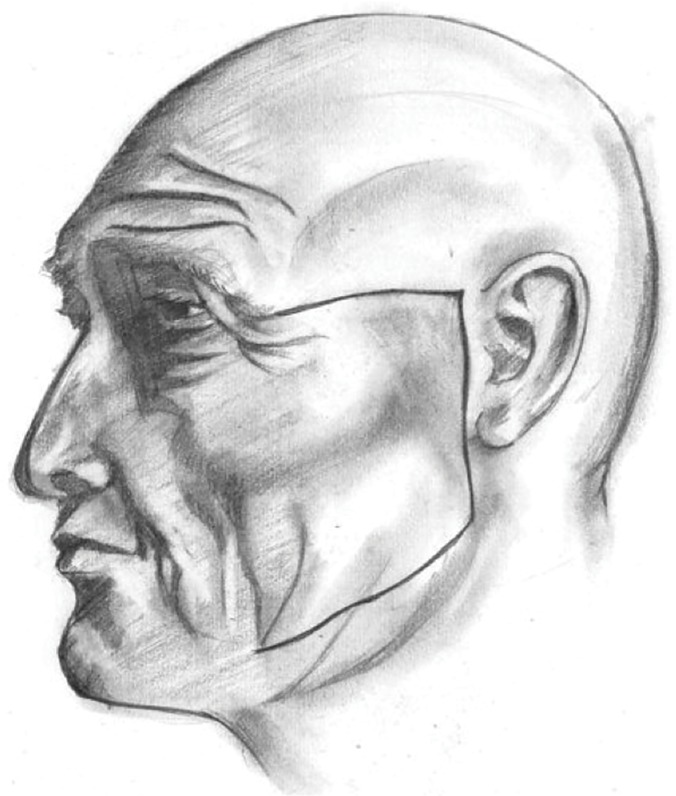
Cutaneous incisions (adult).

**Fig 2:**
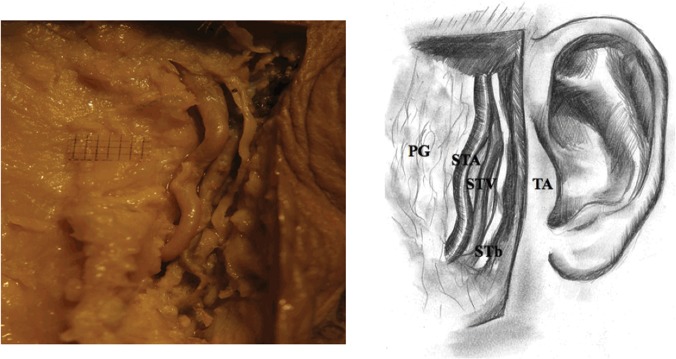
Superficial temporal branch of the auriculotemporal nerve (adult), PG—parotid gland, STA—superficial temporal artery, STV—superficial temporal vein, STb—superficial temporal branch of the auriculotemporal nerve, TA—tragus of the auricle.

**Fig 3:**
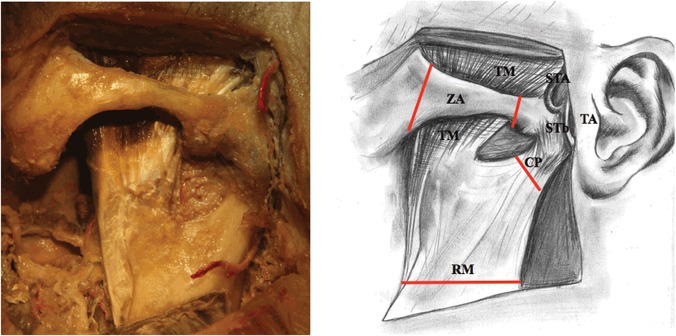
Exposure of the ramus of the mandible and zygomatic arch (adult), STA—superficial temporal artery, TA—tragus of the auricle, TM—temporalis muscle, STb—superficial temporal branch of the auriculotemporal nerve, CP—condylar process of the mandible, RM—ramus of the mandible, ZA—zygomatic arch. Lines of incisions to osseous structures are marked red.

**Fig 4:**
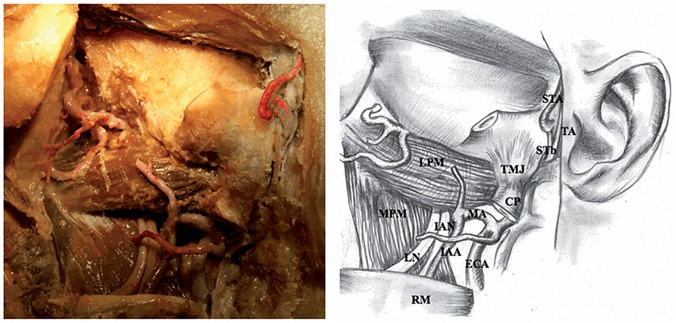
Nerves of pterygomandibular space (adult). STA—superficial temporal artery, TA—tragus of the auricle, STb—superficial temporal branch of the auriculotemporal nerve, LPM—lateral pterygoid muscle, TMJ—temporomandibular joint, CP—condylar process of the mandible, MA—maxillary artery, MPM—medial pterygoid muscle, IAN—inferior alveolar nerve, IAA—inferior alveolar artery, LN—lingual nerve, ECA—external carotid artery, RM—ramus of the mandible.

(i) Nerve preparation in adults: 1) Firstly, two cutaneous incisions were made—two horizontal incisions and one vertical incision. The first horizontal incision was parallel to the superior margin of the zygomatic arch, the second one—parallel to the inferior margin of the body of the mandible. Vertical incision was located anteriorly to the tragus and connected both horizontal incisions (Fig 1).

2) Later, superficial temporal branch of ATN was prepared to the point in which this branch emerges from behind the posterior margin of the neck of the mandible. The aim of preparing the superficial temporal branch at its beginning was to prevent its damage during further stages of preparation. This branch is helpful in later stages to identify the ATN trunk (Fig 2).

3) Further on, a cutaneous flap was elevated and further layers of tissues were prepared until the ramus of the mandible and zygomatic arch were exposed (Fig 3).

4) Zygomatic arch was cut in two places and incisions were made in the neck of the mandible and in the inferior part of the ramus of the mandible. Then, the fragments that had been cut off were removed. Attention should be paid during preparation in order for the cutting tool not to destroy the ATN trunk located medially to the neck of the mandible, and the lingual nerve and the inferior alveolar nerve located medially to the ramus of the mandible. In order not to cut the nerves, a metal spatula was inserted into the pterygomandibular space and it was guided on the medial surface of the ramus of the mandible. 5) Exposure of the lateral surface of the lateral pterygoid muscle and the lateral surface of the medial pterygoid muscle, and the preparation of the lingual nerve and inferior alveolar nerve located on the lateral surface of the medial pterygoid muscle (Fig 4).

**Fig 5:**
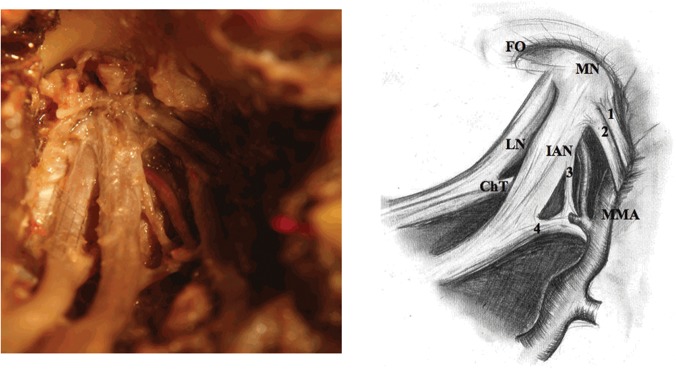
Exposure of the mandibular nerve trunk (adult). 1, 2, 3 and 4—each root of the auriculotemporal nerve, FO—foramen ovale, MN—mandibular nerve, LN—lingual nerve, IAN—inferior alveolar nerve, ChT—chorda tympani, MMA—middle meningeal artery.

During inferior alveolar nerve preparation one should pay attention to the ATN roots which emerge low, in order not to injure them 6) Removal of the lateral pterygoid muscle and exposure of the trunk of the mandibular nerve along with initial fragments of its branches (Fig 5).

**Fig 6:**
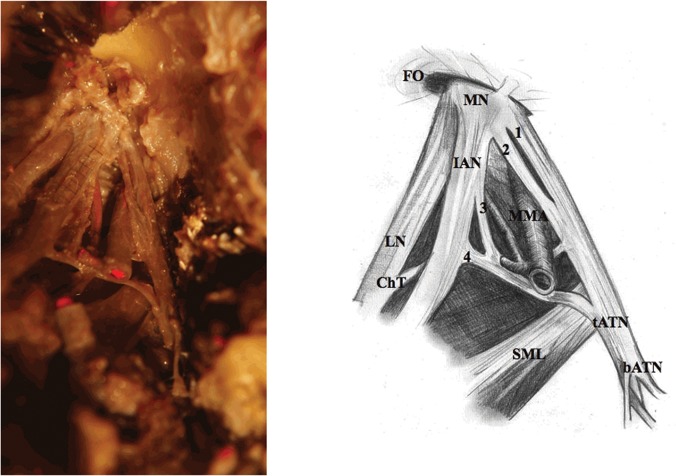
Auriculotemporal nerve (adult). 1, 2, 3 and 4—each root of the auriculotemporal nerve, FO—foramen ovale, MN—mandibular nerve, IAN—inferior alveolar nerve, MMA—middle meningeal artery, LN—lingual nerve, ChT—chorda tympani, tATN—trunk of the auriculotemporal nerve, SML—sphenomandibular ligament, bATN—branches of the auriculotemporal nerve.

7) Preparation of the roots, the trunk and initial fragments of ATN branches (Fig 6).

**Fig 7:**
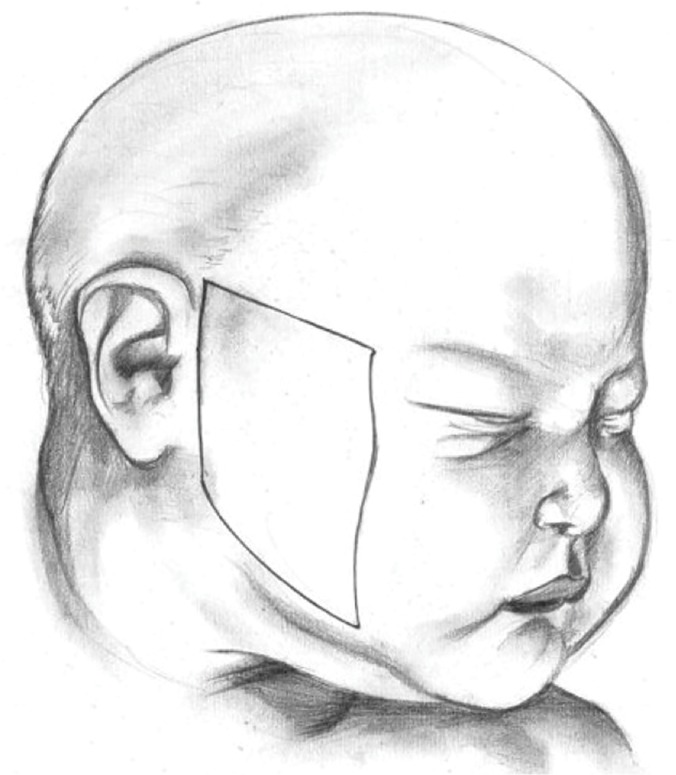
Cutaneous incisions (fetus).

ATN preparation should begin from its lowest roots that originate from the inferior alveolar nerve or mandibular nerve, which enables precise root identification in the points in which they emerge. Exposure of the distal root fragments, various connections between the roots and the trunk of the nerve can be accomplished only after initial parts of all nerve roots have been identified. This enables to prevent injuries to thin inferior nerve roots. 8) ATN collection along with the mandibular nerve and initial parts of the lingual nerve and inferior alveolar nerve. (ii) ATN preparation in fetuses: 1) Creation of two horizontal and two vertical incisions. The first horizontal incision is parallel to the superior margin of the zygomatic arch on the level of the superior margin of the auricle. The second horizontal incision is parallel to the inferior margin of the body of the mandible. The first vertical incision lies anteriorly to the tragus, the second vertical incision is parallel to the first one and is located at the level of the lateral margin of the eye socket. (Fig 7).

**Fig 8:**
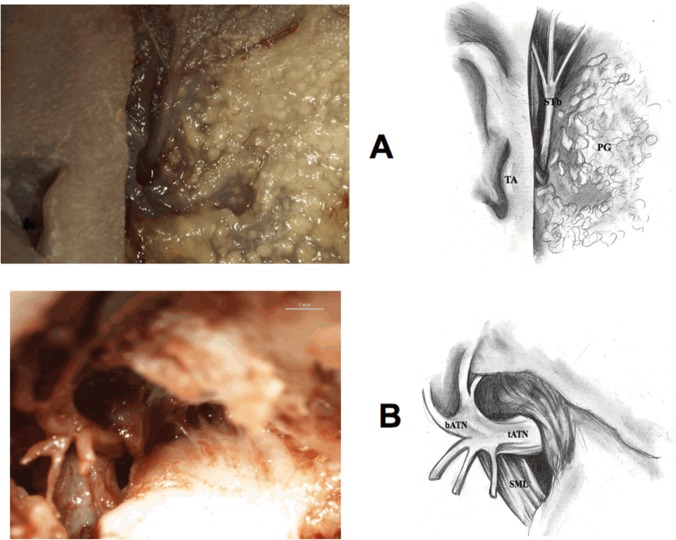
Auriculotemporal nerve preparation (fetus). STb—superficial temporal branch of the auriculotemporal nerve, PG—parotid gland, TA—tragus of the auricle, bATN—branches of the auriculotemporal nerve, tATN—trunk of the auriculotemporal nerve, SML—sphenomandibular ligament.

2) Preparing the superficial temporal branch of ATN to the point in which this branch emerges from the posterior margin of the neck of the mandible. Preparation of the superficial temporal branch enables identification of the nerve trunk in the next stage (Fig 8A). 3) ATN trunk preparation (Fig 8B).

**Fig 9:**
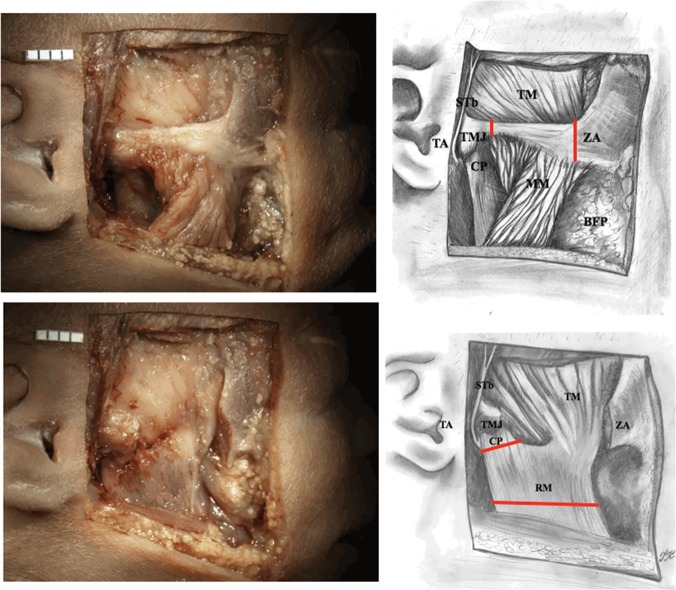
Exposure of the ramus of the mandible and zygomatic arch (fetus). TM—temporalis muscle, STb—superficial temporal branch of the auriculotemporal nerve, TMJ—temporomandibular joint, ZA—zygomatic arch, TA—tragus of the auricle, CP—condylar process of the mandible, MM—masseter muscle, BFP—buccal fat pad, RM—ramus of the mandible. Lines of incisions to osseous structures are marked red.

By preparing the tissues along the superficial temporal branch in the inferior and anterior direction we reach the point in which ATN trunk divides into several branches. This point is located medially and slightly posteriorly to the condyle. We gradually move from the point of division to the main part of the trunk located medially from the neck of the mandible 4) Preparation of further tissue layers in the parotideomasseteric region until the exposure of the ramus of the mandible and temporomandibular joint. 5) Preparation of incisions in the zygomatic arch. Later on, incisions to the condyle and inferior part of the ramus of the mandible are made. Fragments that were cut off are removed (Fig 9).

**Fig 10:**
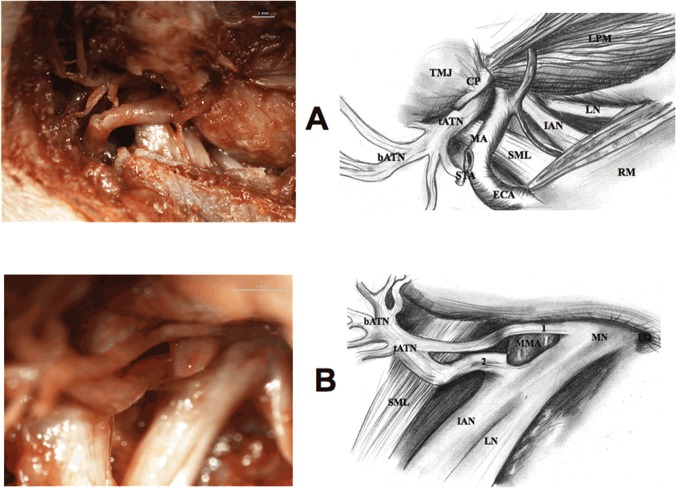
Opening of the infratemporal fossa (fetus). 1 and 2—each root of the auriculotemporal nerve, LPM—lateral pterygoid muscle, LN—lingual nerve, tATN—trunk of the auriculotemporal nerve, IAN—inferior alveolar nerve, MA—maxillary artery, SML—sphenomandibular ligament, bATN—branches of the auriculotemporal nerve, RM—ramus of the mandible, STA—superficial temporal artery (cut-off), ECA—external carotid artery, MN—mandibular nerve, FO—foramen ovale, MMA—middle meningeal artery.

6) Preparation of the inferior alveolar nerve and lingual nerve located on the lateral surface of the medial pterygoid muscle (Fig 10A).

7) ATN root preparation (Fig 10B). ATN root preparation in fetuses was accomplished from the side in which the roots connect into the trunk. The length of ATN structures, also its roots, is small, and connective tissue surrounding the nerve is softer and more prone to preparation in comparison to adult cadavers. This enables easier preparation of nerve roots from the site in which they connect into one common trunk, to the portion located on the posterior margin of the mandibular nerve or inferior alveolar nerve. 8) Collecting ATN together with mandibular nerve, inferior alveolar nerve and lingual nerve.

Photographic documentation was performed during each stage of preparation.

**Fig 11:**
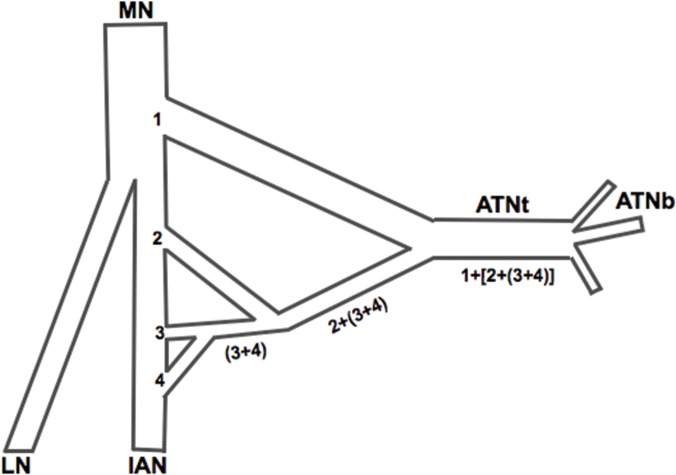
Scheme of nerve structure examination. 1, 2, 3 and 4-each root of the ATN, MN—mandibular nerve, LN—lingual nerve, IAN—inferior alveolar nerve, ATNt—trunk of the auriculotemporal nerve, ATNb—branches of the auriculotemporal nerve, 1, 2, 3 and 4-each root of ATN, (3+4)—secondary roots of ATN, 2+(3+4) tertiary root of ATN, 1+[2+(3+4)]—roots forming the nerve trunk.

Collected nerves: auriculotemporal, mandibular, lingual, inferior alveolar were described in detail and measured according to the presented scheme (Fig 11).

A three-point analysis of the ATN specimens was based on the following criteria:

- the number of roots
- the way of root division
- configuration of the interradicular fibers that form the ATN trunk

A chi-square test for difference in proportions was used in our analysis. Statistical analysis was based on the software R Project for Statistical Computing. All parameters were compared via the chi-square test, with P-values under 0.05 considered statistically significant.

## Results

**Fig 12:**
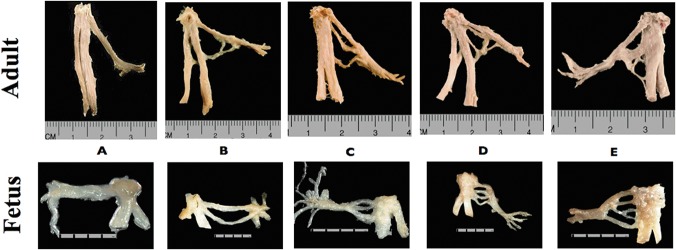
Variants of the auriculotemporal nerve by the number of roots. A—one-root variant, B—two-root variant, C—three-root variant, D—four-root variant, E—five-root variant.

Each ATN revealed one to five roots (Fig 12). A three-root variant was found to be the most common, evident in 32% of all specimens, adult and fetus alike. The next most common variant was the two-root variant, evident in 27% of adult specimens and 32% of the fetal specimens. Specimens exhibiting four and five roots proved to be the most rare. No significant differences were noted when comparing nerve specimens from the left side versus those from the right side of the cadavers (Table 1).

**Table 1:** Total number of each variant of the auriculotemporal nerve in the population studied.

No. of roots	Adult				Fetus				Total			
	L	R	p	Total	L	R	P	Total	L	R	p	Total
1	4/9 (44%)	5/9 (56%)	1	9/40 (22.5%)	3/7 (43%)	4/7 (57%)	1	7/40 (17.5%)	7/16 (44%)	9/16 (56%)	0.8026	16/80 (20%)
2	7/11 (63%)	4/11 (36%)	0.3636	11/40 (27.5%)	8/13 (61%)	5/13 (38%)	0.5791	13/40 (32.5%)	15/24 (62%)	9/24 (38%)	0.3074	24/80 (30%)
3	6/13 (46%)	7/13 (54%)	1	13/40 (32.5%)	4/13 (31%)	9/13 (69%)	0.2673	13/40 (32.5%)	10/26 (38%)	16/26 (61%)	0.3268	26/80 (32.5%)
4	1/1 (100%)	-	1	1/20 (5%)	3/4 (75%)	1/4 (25%)	0.6171	4/40 (10%)	4/5 (80%)	1/5 (20%)	0.3711	5/80 (6%)
5	2/6 (33%)	4/6 (67%)	0.6831	6/40 (15%)	1/3 (33%)	2/3 (67%)	1	3/40 (7.5%)	3/9 (34%)	6/9 (67%)	0.505	9/80 (11%)

During study of the way of root division, the second parameter under investigation, it was observed that approximately 50% of both adult and fetus specimens exhibited ATN roots that originated exclusively from mandibular nerve (MN) (19:40 and 22:40, respectively). In the remaining cases, ATN roots originated from MN and the inferior alveolar nerve (IAN).

**Fig 13:**
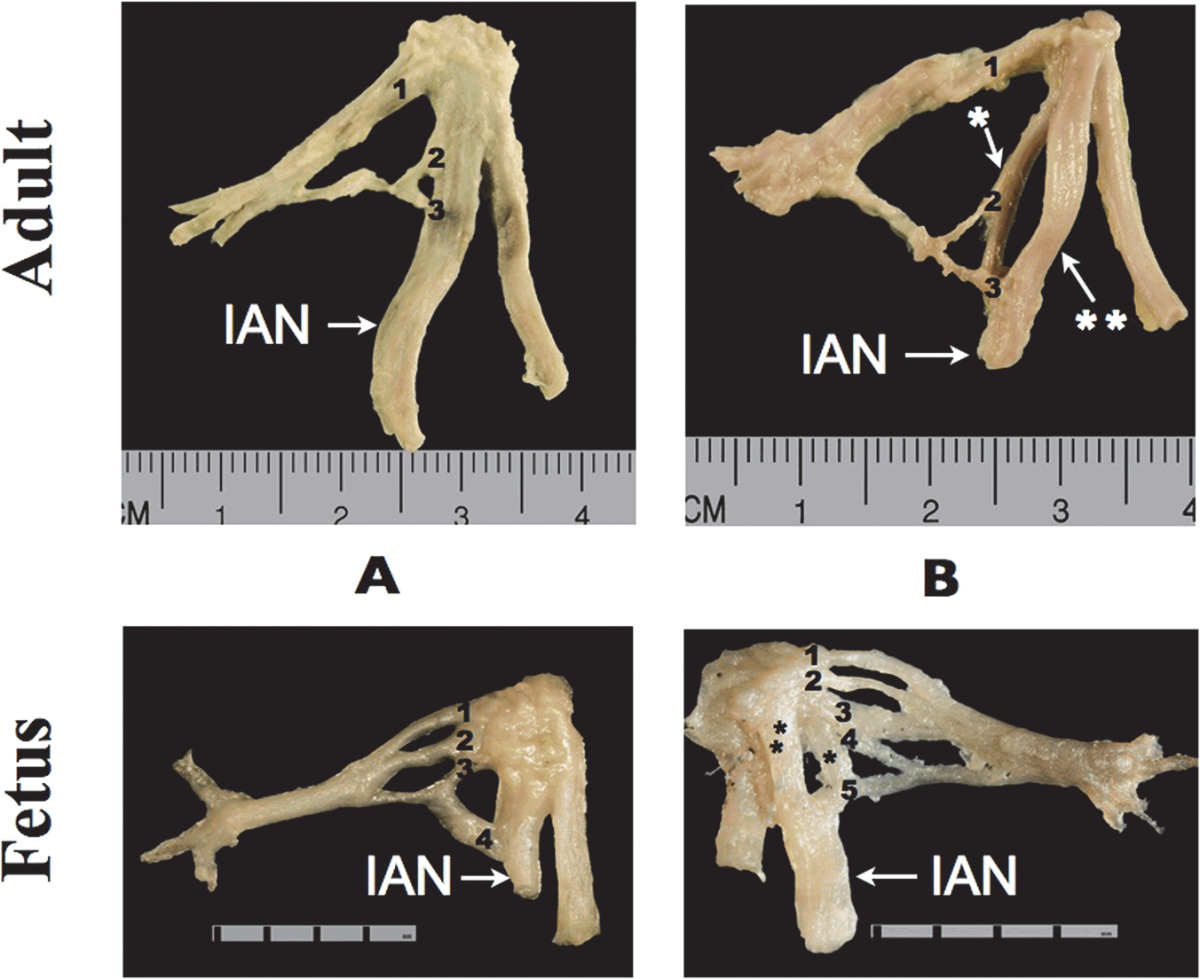
The way of root division in adults and fetuses. Adult A and B—three-root variant (R); Fetus A—four-root variant (R), Fetus B—five-root variant (L). 1, 2, 3, 4 and 5—each root of the auriculotemporal nerve, IAN—inferior alveolar nerve; * posterior ramus of the trunk of the inferior alveolar nerve, ** anterior ramus of the trunk of the inferior alveolar nerve.

Furthermore, two variants of IAN were observed: bifurcated and non-bifurcated. In the bifurcated variant, the trunk of the nerve is divided into two rami: anterior and posterior. This finding led to categorization of the IAN-originating ATN roots into two sub-groups: root variants that originated from bifurcated IANs and root variants that originated from non-bifurcated IANs (Fig 13A—adult: root 2 and 3; fetus: root 4). It was noted that ATN roots involving bifurcated IANs arose at both the anterior (Fig 13B—adult: root 3) and posterior (Fig 13B—adult: root 2; fetus: root 3, 4, and 5) rami of the trunk (Table 2).

**Table 2:** Variants of auriculotemporal nerve roots by the way of root division (the start-point of the root). MN—mandibular nerve, ATN—auriculotemporal nerve.

No. ATN roots	Adult					Fetus				
	MN	Inferior alveolar nerve				MN	Inferior alveolar nerve			
		non-bifurcated#	bifurcated#		Total No of variants arising from IAN		non-bifurcated#	bifurcated#		Total No of variants arising from IAN
			posterior rami	anterior rami				posterior rami	anterior rami	
1	6/19 (31%)	3/17 (18%)	0/4 (-)	0/1 (-)	3/21 (14.3%)	6/22 (27.3%)	1/17 (5.9%)	0/0 (-)	0/0 (-)	1/18 (5.5%)
2	7/19 (37%)	4/17 (23.5%)	0/4 (-)	0/1 (-)	4/21 (19.0%)	11/22 (50%)	2/17 (11.8%)	0/0 (-)	0/0 (-)	2/18 (11.1%)
3	4/19 (21%)	6/17 (35.3%)	3/4 (75%)	0/1 (-)	9/21 (42.8%)	3/22 (14%)	10/17 (58.8%)	0/0 (-)	0/0 (-)	10/18 (55.5%)
4	1/19 (5.2%)	0/17 (-)	0/4 (-)	0/1 (-)	0/21 (-)	1/22 (4.5%)	3/17 (17.6%)	0/0 (-)	0/0 (-)	3/18 (16.7%)
5	1/19 (5.2%)	4/17 (23.5%)	1*/4 (25%)	1*/1 (100%)	5/21 (23.8%)	1/22 (4.5%)	1/17 (5.9%)	1/1 (100%)	0/0 (-)	2/18 (11.1%)

* root variants that belong to the same nerve.

# start point on the trunk of the nerve

Further observation revealed that inferior origin of ATN roots was frequently associated with a multi-root structure of the nerve, most notably in three-, four-, and five-root variants. This discovery led to the development of start-point sub-categories:

- Low-rooted, consisting of one or two roots
- High-rooted, consisting of three, four, or five roots

**Fig 14:**
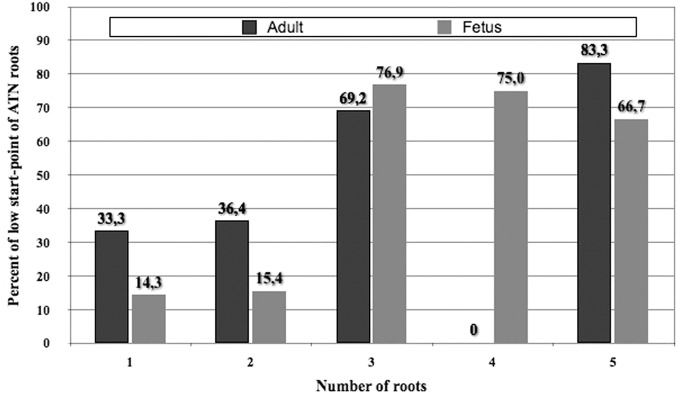
Percentage of inferior roots (connecting to IAN) in each variant of the auriculotemporal nerve.

A significant difference was noted between the high-lying root and low-lying root groups according to the origin of inferior ATN roots (χ2 = 16.210, P <0.0001), irrespective of the adult or fetus source of the dissected specimen. In the fetus population, a significant difference was also observed (χ2 = 12.222, P = 0.0005), while the difference in the adult population was not significant (χ2 = 2.386, P = 0.1224) (Fig 14).

The trunk of ATN was found to display many different configurations, leading to the third aspect of the study: configuration of interradicular fibers and the final number of roots that form the ATN trunk. Regardless of the number of roots joining together to form ATN, their interradicular fibers most often led to two primary root formations. Finally, those two primary, or main, roots united to create the trunk of ATN in 63 of the 80 (78.8%) cases under study. It was observed that the trunk is most rarely formed from 3 roots (in 1.3% of cases). In the remaining 16 cases (20%), the trunk was formed from one root (Table 3).

**Table 3:** Configuration of interradicular fibers and trunk formation.

Number of primary roots	Configuration of interradicular fibers	Number of roots reaching the trunk	Number of variants					
			Adult			Fetus		
			R	L	Total	R	L	Total
1	1	1	5	4	9	4	3	7
2	1*+2*	2	4	7	11	5	8	13
3	1*+2*+3*	3	0	0	0	0	1	1
	1* +(2+3)	2	5	2	7	4	1	5
	3* +(1+2)	2	1	4	5	4	1	5
	3b* +(1+2+3a)	2	0	0	0	0	1	1
	(1+2a)+(2b+3)	2	0	0	0	1	0	1
	2* +(1+3)	2	1	0	1	0	0	0
4	(1+2)+(3+4)	2	0	1	1	1	1	2
	4* + (1+2+3)	2	0	0	0	0	1	1
	1* +[2+(3+4)]	2	0	0	0	0	1	1
5	1* +[(2+3)+(4+5)]	2	2	0	2	0	0	0
	1* +[2+(3+4+5)]	2	2	1	3	0	0	0
	(1+2+3)+(4+5)	2	0	0	0	0	1	1
	5* +[(1+2)+(3+4)]	2	0	0	0	2	0	2
	1*+ [(2+4)+(3+5)]	2	0	1	1	0	0	0

*roots independently reaching the trunk.

Configurations of roots in high-rooted variants were systematized:

- Three-rooted variants exhibited six configurations
- Four-rooted variants exhibited three configurations
- Five-rooted variants exhibited five configurations

**Fig 15:**
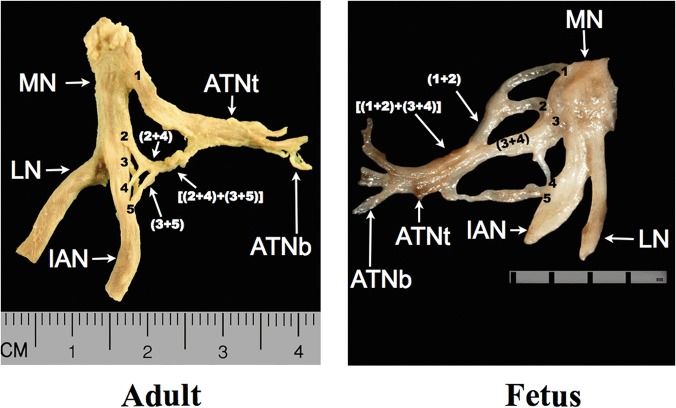
Configuration of interradicular fibers in the auriculotemporal nerve (ATN). Adult, five-root variant (L). Fetus, five-root variant (R). 1, 2, 3, 4 and 5—each root of ATN, (n+n)—secondary roots of ATN, [(n+n)+(n+n)]—tertiary root of ATN; ATNt—trunk of ATN, ATNb—branch of ATN, MN—mandibular nerve, LN—lingual nerve, IAN—inferior alveolar nerve.

In each configuration, the primary roots connect with each other to form secondary and tertiary roots. In the adult specimen in Fig 15, for example:

- Root 1 does not form interradicular connections
- Roots 2 and 4 form a secondary root (2+4)
- Roots 3 and 5 form the other secondary root (3+5)
- Both secondary roots (2+4) and (3+5) connect to form a tertiary root [(2+4)+(3+5)]

The tertiary root [(2+4)+(3+5)] then connects to root 1 to form the ATN trunk

In the Fig 15 fetus example:

- Roots 1 and 2 form a secondary root (1+2)
- Roots 3 and 4 also form a secondary root (3+4)
- Both secondary roots connect to form a tertiary root [(1+2)+(3+4)]
- This tertiary root [(1+2)+(3+4)] connects to root 5 to form the nerve’s trunk

In both three-root variants, it was observed that the primary roots (2 and 3) divide into secondary roots (a and b).

## Discussion

The diversity of the ATN structure has been previously known and widely described in literature. In 1956, Krizan was the first to classify ATN by its location in relation to MMA. Four types of nerves were distinguished in this work. According to Krizan’s classification:

- Type I and IV, consisting of two-root variants with a blood vessel in between (vessels in type I were MMA and maxillary artery [MA] while in type IV, it was only MA)
- Types II and III, consisting of one-root nerve located medially to MA (type II) or laterally to MMA (type III).

Two decades later, in 1971, Baumel distinguished 12 variants of ATN [11]. Classification criteria were as follows: the number of roots, root length, and its relation to MMA. Finally, in 2005, Gülekon proposed the number of roots to be the main criterion of classification, which has become helpful [12]. It is important to bear in mind, that this work focuses on the nerve itself and omits the issue of the root division and configurations of interradicular connections. The study by Dias is also noteworthy [10]. The author proposed a 10-variant classification system that comprises both the way of ATN root division and the relationship between nerve roots and MMA; not taking into account the complicated configuration of interradicular connections.

That is why based on the findings of this study, it is recommended that the classification of ATN should be based on three criteria:

- the number of roots,
- the way of root division, and
- configuration of the interradicular connections.

(i.) The Number of Roots—Historically, the literature on the infratemporal section of ATN identifies equivalently used terms, such as, root [5, 11–13, 15] and branch [9, 16, 17] to name the ramus of the nerve. The reason of this equivalency is that ATN roots could start on MN as well as on IAN. Scientists often classify the low-start roots of ATN as branches connecting the nerve with IAN. The first argument for calling the inferior ramus of ATN a “root” is the fact that in some one-root variants (33.3% of adults and 14.3% of fetuses) only a part of the root of the nerve originates from MN and IAN (Fig 14). If one accepted the assumption that fibers, arising from IAN and passing to ATN, could be considered as “connecting” the nerve components, the superior part of the root would represent ATN and the inferior would resemble a branch of IAN. Previous analyses of the anatomy of IAN introduce the term “connecting branches” to name the structure of inferior roots of ATN, which connect IAN with ATN [16, 17]. However, if such a nomenclature was accepted in the multi-root variants, one would need to consequently use it for one-root variants, a practice considered quite controversial.

The second reason for using the term “root” is the fact that generally in multi-root variants, most of the roots arise from IAN (Fig 12D, adult).

In such instances MN is commonly divided into IAN and the lingual nerve, while low-lying roots are an integral part of the ATN root system. From the point of view of IAN anatomy, it could be partially accepted that the low-lying ATN roots play a role of connecting IAN with ATN. However, considering the anatomy of ATN, these are still its roots. An analogy is known in anatomy of the brachial plexus, where its roots are branches of the C5-Th1 abdominal ganglia [18].

Taking those arguments into account, it is suggested to distinguish 5 variants of ATN structure based on the presented research: (i) one-, (ii) two-, (iii) three-, (iv) four-, and (v) five-root variants.

Commonly, authors describe one-root [12] or two-root variants [5, 9, 10, 11, 13] while multi-root variants are rarely noted. The four-root variant, documented exclusively by Baumel [11], Gülekon [12] and Dias [10] was distinguished as a single case. The five-root variant had not been described previously although it was found to exist in 11% of the specimens examined in this study. Importantly, this variant was observed in both adults and fetuses beyond 18 weeks of gestation (Table 1), indicating that the formation of the nerve rami occurs in the early phase of ontogenesis.

(ii.) The Way of Root Division—In the current study, two forms of root division were distinguished: (I) roots that arise from MN and (II) roots that arise from IAN. As a bifurcated type of IAN occurs, roots originating from IAN (II) can be distinguished by two sub-types: II-1, in which the roots arise from non-bifurcated IAN and II-2, in which the roots arise from bifurcated IAN. In the second type, two further sub-types can be distinguished: II-2-a, in which the roots arise from the posterior ramus of the trunk of IAN; and II-2-b, in which the roots originate in the anterior ramus of the IAN trunk.

(iii.) Configuration of the Interradicular Fibers Forming the Main Nerve Trunk—A visible tendency to form a net of interradicular fibers in multi-root variants was observed. Rare secondary roots are the effects of these connections. Secondary roots connect to form the main trunk. Such a tendency was noted in adult as well as fetus specimens. This finding prompts the recommendation to distinguish 3 conformations: (1) the trunk is formed from one root; (2) the trunk is formed from two roots, and (3) the trunk is formed from three roots.

The recommendation regarding the structure of the infratemporal section of the ATN is both practical and theoretical. Firstly, it is particularly important to predict the variability of ATN anatomy in the field of surgery involving the infratemporal fossa and dental anesthesia. Specifiaclly, correct recognition of these structures could prove highly advantageous in the surgical field [19–25] as well as demystifying possible side effects that occur during administration of local anesthesia to IAN [26–27]. Secondly, awareness of ATN anatomy seems to be particularly useful in understanding and explaining the mechanism of neuralgia [28–30] and migraine headaches [8]. The standardization of nomenclature recommended as a result of this study will advance understanding of the anatomy of ATN; it will also bring a new dynamic to the variability of structures in such a fascinating location as the infratemporal fossa.

## Conclusions

In conclusion to this investigation, the findings are summarized as:

1. There were no significant differences between the specimens derived from the adults and fetuses.
2. There were no significant differences between specimens derived from the representatives of different age and sex.
3. Five variants of the nerve could be classified according to the number of ATN roots: one-, two-, three-, four- and five-root variants.
4. The roots of ATN could start from MN or from IAN. In the occurrence of a bifurcated type of IAN, ATN roots could start from the anterior or posterior ramus of the IAN trunk.
5. Irrespective of the number of primary roots, the trunk is finally formed from one, two or three roots.
